# Characterization of white spot lesions formed on human enamel under microcosm biofilm for different experimental periods

**DOI:** 10.1590/1678-7757-2021-0560

**Published:** 2022-04-01

**Authors:** Flávia Mauad Levy, Aline Silva Braga, Vinícius Taioqui Pelá, Stacey Lavender, Dennis Zhang, Shira Pilch, Zilson Malheiros, Bernal Stewart, Ana Carolina Magalhães, Marília Afonso Rabelo Buzalaf

**Affiliations:** 1 Universidade de São Paulo Faculdade de Odontologia de Bauru Departamento de Ciências Biológicas Bauru Brasil Universidade de São Paulo, Faculdade de Odontologia de Bauru, Departamento de Ciências Biológicas, Bauru, Brasil.; 2 Colgate Palmolive Company Global Technology Center Piscataway New Jersey USA Colgate Palmolive Company, Global Technology Center, Piscataway, New Jersey, USA.

**Keywords:** Bacteria, Biofilm, Enamel

## Abstract

The initial characteristics of white spot lesion (WSLs), such as the degree of integrated mineral loss (ΔZ), depth and pattern of mineral distribution, have an impact on further demineralization and remineralization. However, these lesion parameters have not been evaluated in WSLs produced from microcosm biofilms.

**Objective:**

This study characterized artificial white spot lesions produced on human enamel under microcosm biofilm for different experimental periods.

**Methodology:**

In total, 100 human enamel specimens (4x4mm) were assigned to 5 distinct groups (n=20/group) differing according to the period of biofilm formation (2, 4, 6, 8 or 10 days). Microcosm biofilm was produced on the specimens from a mixture of human and McBain saliva at the first 8h. Enamel samples were then exposed to McBain saliva containing 0.2% sucrose. WSLs formed were characterized by quantitative light-induced fluorescence (QLF) and transverse microradiography (TMR). Data were analyzed by ANOVA/Tukey or Kruskal-Wallis/Dunn tests (p<0.05).

**Results:**

A clear time-response pattern was observed for both analyses, but TMR was able to better discriminate among the lesions. Regarding QLF analysis, median (95%CI; %) changes in fluorescence ∆Z were -7.74(-7.74:-6.45)^a^, -8.52(-8.75:-8.00)^ab^, -9.17(-10.00:-8.71)^bc^, -9.58(-10.53:-8.99)^bc^ and -10.01(-11.44:-9.72)^c^ for 2, 4, 6, 8, and 10 days, respectively. For TMR, median (95%CI; vol%.µm) ∆Z were 1410(1299-1479)^a^, 2420(2327-2604)^ab^, 2775(2573-2899)^bc^, 3305(3192-3406)^cd^ and 4330(3972-4465)^d^, whereas mean (SD; µm) lesion depth were 53.7(12.3)^a^, 71.4(12.0)^a^, 103.8(24.8)^b^, 130.5(27.2)^bc^, 167.2(39.3)^c^ for 2, 4, 6, 8 and 10 days, respectively.

**Conclusion:**

The progression of WSLs formed on human enamel under microcosm biofilm can be characterized over 2-10 days, both by QLF and TMR analyses, although the latter provides better discrimination among the lesions.

## Introduction

Worldwide, dental caries is the most prevalent chronic disease, being considered an important public health problem.^1^ The lesion results from the development of a cariogenic biofilm due to the interaction, along time, of frequent sugar consumption, poor oral hygiene, and unfavourable host factors.^2,3^ The first clinical sign of dental caries is a white spot lesion (WSL), that is characterized histologically by a subsurface demineralization below a pseudo-intact outermost enamel layer. At this stage, the lesion can still be remineralized. For this reason, several methodologies have been proposed to create artificial WSLs to delevop and evaluate new therapeutic approaches.

While for natural WSLs the presence of a biofilm is essential to develop the lesion, artificial lesions can be created using abiotic models, involving demineralizing agents or pH-cycling protocols^4^. Biotic *in vitro* models include single species, multi-species or a microcosm approach.^5^ Microcosm biofilms that originate from the whole-mixed natural microbiota have the important advantage of representing the natural microbiota in its entirety, allowing the replication of the complex interactions within the oral ecosystem.^6,7^ Moreover, microcosm biofilms are not steady-state systems, e.g. they evolve, thus resembling dental plaque *in vivo*.^5^

Artificial WSLs are typically produced to test the efficacy of anti-caries products.^4^ Thus, the initial characteristics of the lesions, such as the degree of integrated mineral loss (ΔZ), depth and pattern of mineral distribution, have an impact on further demineralization and remineralization,^8-11^ which can affect the performance of the product that is being tested. However, these lesion parameters have not been evaluated in WSLs produced from microcosm biofilms. We raised the hypothesis that, by varying the experimental time, it is possible to produce distinct lesions in terms of mineral loss and depth, with different abilities to respond to the action of distinct remineralizing products. Thus, this study aimed to characterize artificial WSLs produced on human enamel under microcosm biofilm for different experimental periods.

## Methodology

### Study design

**Figure 1:**
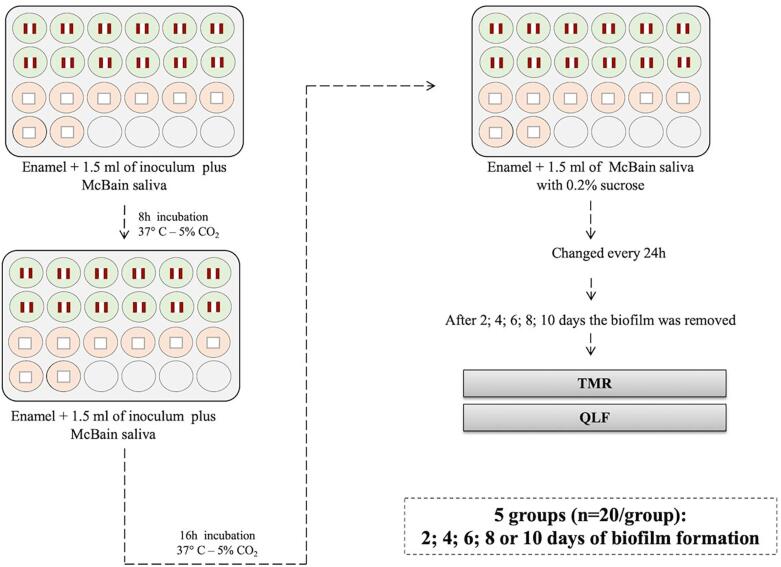
Experimental protocol

The protocol of this study was approved by the local ethical committee (CAAE 99086718.0.0000.5417). Saliva was collected from ten volunteers. In total, 100 enamel specimens were obtained from unerupted third molars and divided into 5 groups, according with the period of biofilm formation (2, 4, 6, 8 or 10 days). Microcosm biofilm was produced on the specimens from a mixture of human and McBain saliva at the first 8 h. Enamel samples were then exposed to McBain saliva containing 0.2% sucrose. WSLs formed were characterized by quantitative light-induced fluorescence (QLF) and transverse microradiography (TMR) (Figure 1).

### Saliva collection

After informed consent was provided, saliva was collected from 10 healthy donors (22-35 years old) who met the inclusion criteria: (1) normal salivary flow (stimulated salivary flow > 1 ml/min and non-stimulated salivary flow > 0.3 ml/min), (2) caries history but no active caries (no active white-spot and/or cavitated lesions); (3) absence of gingivitis/periodontitis (gum bleeding or tooth mobility); and (4) no ingestion of antibiotics for three months prior to the experiment. The exclusion criteria were the conditions opposite to those showed above, as well as individuals with chronic systemic diseases, smokers, pregnant, and lactating women. Prior to the day of collection, the donors did not brush their teeth. Furthermore, they were not allowed to ingest food or drinks within the last 2h before saliva collection. The saliva was collected under stimulation by chewing a rubber material for 10 min in the morning. After collection, saliva was pooled and diluted in glycerol (70% saliva and 30% glycerol). Aliquots of 1 ml were stored at - 80°C.^12-14^

### Specimen preparation

For the analysis, 100 human enamel specimens (4x4 mm) were prepared from recently extracted unerupted third molars, previously stored in 0.1% thymol pH 7.0, using a semi-precision cutting machine (Buehler, Enfield, CT, USA). The samples were fixed in acrylic disks with wax and polished in a metallographic polishing machine (Arotec, Cotia, SP, Brazil) using water-cooled silicon-carbide disks (600-grade papers ANSI grit; Buehler) to remove grooves and to standardize the surface roughness (0.150 ± 0.040 μm as the mean and SD). Mean surface roughness (Ra) was assessed using a contact profilometer and Mahr Surf XCR 20 software (Mahr, Göttingen, LS, Germany), for randomization purposes. Roughness was applied to standardize the tooth surface for biofilm growth, allowing the groups to show similar surface roughness values before biofilm growth.^12^ In half of the specimens, two-thirds of the enamel surfaces were protected with nail polish to obtain control areas for the transverse microradiography (TMR) analysis. The samples were sterilized with ethylene oxide (gas exposure time [30% ETO/ 70%CO_2_] for 4 h under 0.5±0.1 kgF cm^-2^ in pressure).

Based on their Ra values, the specimens were divided into 5 groups that differed regarding the period of microcosm biofilm formation: 2, 4, 6, 8 and 10 days (n=20/group; 10 for QLF and 10 for TMR analysis).

### Microcosm biofilm formation

The human saliva was defrosted and mixed with McBain artificial saliva^15^ in the proportion of 1:50. The McBain saliva contained 2.5 g l^−1^ of mucin from porcine stomach (type II), 2.0 g l^−1^ of bacteriological peptone, 2.0 g l^−1^ of tryptone, 1.0 g l^−1^ of yeast extract, 0.35 g l^−1^ of NaCl, 0.2 g l^−1^ of KCl, 0.2 g l^−1^ of CaCl_2_, 0.1 g l^−1^ of cysteine hydrochloride, 0.001 g l^−1^ of hemin and 0.0002 g l^−1^ of vitamin K1, at pH 7.0. All reagents were from SigmaAldrich (St. Louis, MO, USA).

The solution of human saliva and McBain saliva was added to each well containing an enamel sample (*v*=1.5 ml well^−1^) in a 24-well plate, which was incubated at 5% CO_2_ and 37°C. After 8 h, the medium was removed, the enamel samples were washed using PBS (Phosphate Buffered Saline) (5s) and fresh McBain saliva now containing 0.2% sucrose was added to the wells (*v*=1.5 ml well^−1^). The microplates were incubated at 5% CO_2_ and 37°C for another 16h, completing the first day. Every 24 h, the medium was changed and incubated in the same conditions as described above until completing 2, 4, 6, 8 or 10 days of culture^12,16^ (Figure 1).

### Quantitative Light-Induced Fluorescence

QLF was applied to measure the changes in the enamel fluorescence. A xenon arc lamp was used as a light source, and an optical filter system, producing blue light with a maximum wavelength of 370 nm, was connected to the microscope by a liquid light guide (Inspektor Research Systems BV, Amsterdam, The Netherlands). The emitted fluorescence by the tooth was collected with a charged coupled device (CCD)-video microcamera (Panasonic WV-KS 152, Matsushita Electric Industrial Co, Ltd, Osaka, Japan) equipped with high pass yellow filter (γ>520 nm) to exclude any excitation or ambient light that might reach the detector, and a special dental mirror to reflect the image of the lesion connected to the camera.

After drying the sample surface (for 5s), images were obtained by QLF, in a completely dark environment. A computer program (Software Inspektor QLF 2.00f; Inspektor Research System BV, Amsterdam, The Netherlands) was used to display, store, browse, and analyze the images. The QLF parameters were: 1) area of the lesion (white spot area [WS], mm^2^) that was the sum of all points within the lesion with fluorescence loss > 5%; and 2) the mean fluorescence loss (ΔF, %, detection threshold of 5%).^17^ The QLF analysis was performed on each sample at baseline and after the formation of WSLs (2, 4, 6, 8 or 10 days). A recent study showed a good sensitivity (0.72–0.91) and specificity (0.74–0.96) of QLF for dental caries detection in primary teeth.^18^ However, there is no information for such parameters in *in vitro* conditions.

### Transverse microradiography

After cleaning, all enamel samples were transversally sectioned and polished to obtain slices 80–100 μm in thickness. The enamel slices were fixed in a sample-holder together with an aluminum calibration step wedge with 14 steps. A microradiograph was taken using an X-ray generator (Softex, Tokyo, Japan) on the glass plate at 20 kV and 20 mA (at a distance of 42 cm) for 13 min. The glass plates were developed for 7 min, rinsed in deionized water, fixed for 7 min in a dark environment and then rinsed in running water for 10 min and air dried (all procedures were performed at 20°C). The developed plate was analyzed using a transmitted light microscope fitted with a 20× objective (Zeiss, Oberkochen, BW, Germany), a charge-coupled device camera (CCD, Canon, Tokyo, Japan), and a computer. Two images per sample were obtained using data acquisition (version 2012) and interpreted using calculation (version 2006) software from Inspektor Research System (Amsterdam, The Netherlands)^12^. Mineral content was calculated based on the study of Angmar, et al.^19^ (1963), assuming the density of the mineral as 3.15 kg l^−1^ and 87 vol% of mineral content for the sound enamel. In addition, the lesion depth (LD, μm), the integrated mineral loss (∆*Z*, vol% μm), and the average mineral loss over the lesion depth (*R*, vol%) were measured .

### Statistical analysis

Data were statistically analyzed using software GraphPad InStat for Windows (GraphPad Software, San Diego, CA, USA). The normal distribution and homogeneity were checked using the Kolmogorov–Smirnov test and Bartlett’s test, respectively. Regarding QLF parameters, WS were analyzed by ANOVA/Tukey’s test and ∆F was compared by Kruskal-Wallis/Dunn´s test. For TMR, ∆Z data were analyzed by Kruskal-Wallis/Dunn´s test, whereas LD data were compared by ANOVA/Tukey’s test. The level of significance was set at 5%.

## Results

Regarding QLF analysis, the median ΔF significantly increased over time (KW=34.838, p<0.0001). The values obtained for 2 days were significantly different than those obtained for 6, 8 and 10 days, while the values found for 4 days significantly differed from those found for 10 days. The other differences were not significant. The mean lesion area also significantly increased over time (F=146.38, p<0.0001), but the difference was only significant between 2 days and the other experimental periods, which did not significantly differ from each other (Table 1).

**Table 1:** Median (95% CI) changes in fluorescence (ΔF, %), mean (SD) lesion area (LA) (mm^2^) measured by quantitative light-induced fluorescence (QLF), mean lesion depth (LD), median (95% CI) integrated mineral loss (ΔZ, vol%.µm) and mean (SD) mineral loss over the lesion depth (LD) of human enamel under microcosm biofilm for different periods

Experimental periods	ΔF (%)	LA (mm2)	LD (µm)	ΔZ (vol%. μm)	R (vol%)
2 days	-7.74 (-7.74: -6.45)^a^	0.04 (0.05)^a^	53.7 (12.3)^a^	1410 (1299:1479)^a^	26.8 (6.8)^a^
4 days	-8.52 (-8.75: -8)^ab^	1.23 (0.2)^b^	71.4 (12)^a^	2420 (2327:2604)^ab^	28.5 (9.3)^a^
6 days	-9.17 (-10.00: -8.71)^bc^	1.40 (0.31)^b^	103.8 (24.8)^b^	2775 (2573:2899)^bc^	30.5 (11.7)^a^
8 days	-9.58 (-10.53: -8.99)^bc^	1.85 (0.38)^b^	130.5 (27.2)^bc^	3305 (3192:3406)^cd^	24.2 (5.9)^a^
10 days	-10.01 (-11.44: -9.72)^c^	2.26 (0.35)^b^	167.2 (39.3)^c^	4330 (3972:4465)^d^	25.2 (6.4)^a^

* Distinct lowercase letters in the same column indicate significant differences among the lesions formed in different periods. Data were analyzed by ANOVA/Tukey (LA, LD and R) or Kruskal-Wallis/Dunn´s tests (ΔF). p<0.05. n=10.

**Figure 2:**
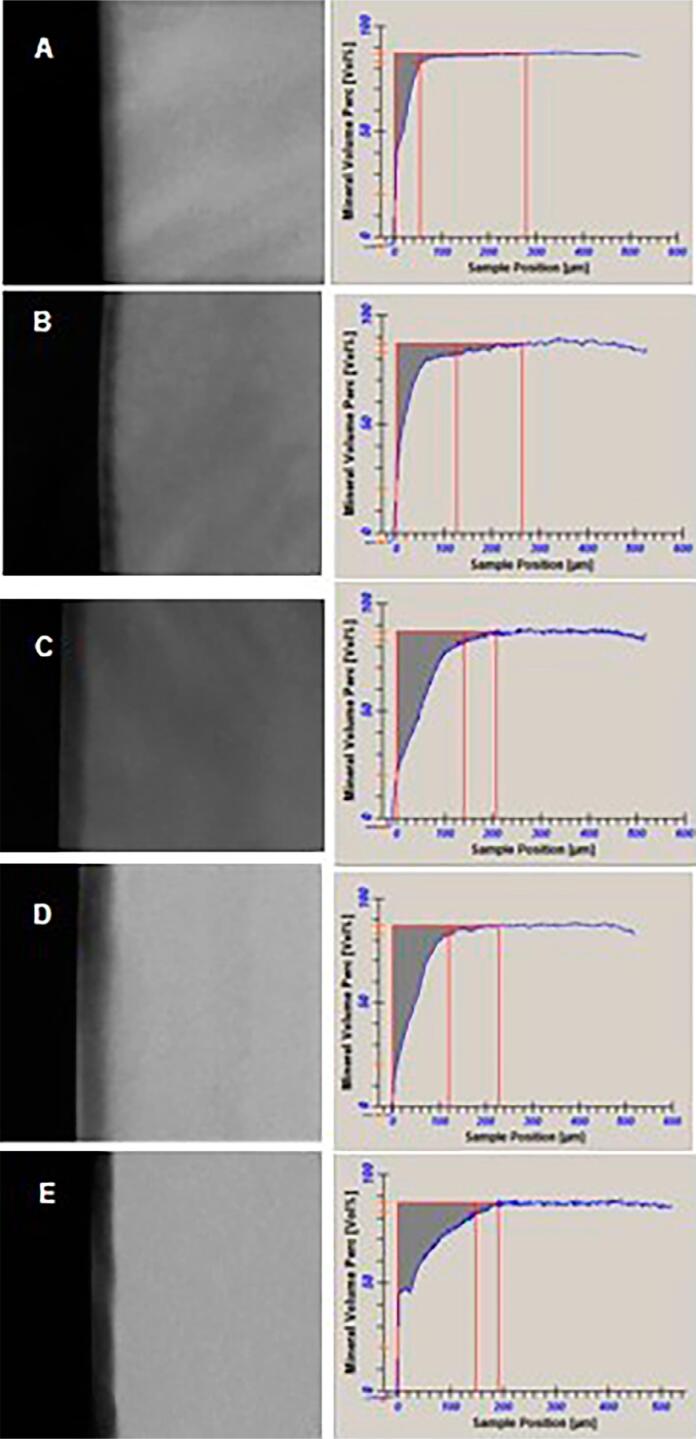
Representative TMR profile of the WSLs produced on enamel under microcosm biofilm for 2 (A), 4 (B), 6 (C), 8 (D) and 10 (E) days. (20X)

A clearer time-response pattern was observed for TMR, which was able to better discriminate among the lesions produced in different time periods when compared with QLF. For ∆Z, Kruskal-Wallis test found a significant difference among the groups (KW=54.591, p<0.0001). Median ∆Z for 2 days was significantly lower than that obtained for 6, 8 and 10 days. Median ∆Z for 4 days was significantly lower than that found for 8 and 10 days and also for 6 days was lower than for 10 days. The other differences were not significant. Regarding lesion depth, ANOVA found a significant difference among the groups (F=42.892, p<0.0001). Lesions formed for 2 and 4 days had similar depths that were significantly lower than the ones found for the formed from 6 days on. Lesions formed for 6 days had depth values significantly lower than those of the lesions formed for 10 days. The other differences were not significant. The pattern of mineral distribution within the lesions formed in the different experimental periods was similar and no significant differences among the mean R values were detected (F=0.865, p=0.491) (Table 1). Figure 2 shows the representative TMR radiographs of the lesions formed in each experimental period.

## Discussion

In our study, we employed a microcosm biofilm model to produce WSLs with different degrees of mineral loss and depth, based on the number of experimental days to which the enamel specimens were subejcted to the microcosm biofilms. This type of biofilm represents the natural microbiota in its entirety,^20^ offering the advantage of replicating the complex interactions within the oral ecosystem.^6,7^ Furthermore, its advantage compared to other *in vitro* caries models, such as pH-cycling model, is that we can study the antibacterial effect of anticaries agents. Then, we can consider microcosm biofilm as a pre-clinical model.^21,22^

In the model, 0.2% sucrose was continuously available in McBain´s artificial saliva from 8 h of the beginning of the experiment.^15^ This procedure was different from other models that employed shorter periods of exposure to higher sucrose concentrations,^23-25^ in which microcosm biofilm was grown under continuous 0.2% sucrose exposure. We evaluated intermittent exposures to 1% sucrose; however, we were unable to induce dental caries lesion formation in 5 days of biofilm growth. Therefore, we kept the continue exposure to sucrose, although this approach does not simulate the *in vivo* intermittent exposure to sugar from a regular diet.

This model has been applied by our research group since 2018.^14^ The presence of sucrose selects cariogenic bacteria in microcosm biofilm, causing pH reduction, which can vary from 4.1 to 5.65 (5 days) under anaerobic and aerobic conditions.^26^ Furthermore, the biofilm microcosm allows growth of cariogenic bacteria such as *Streptococcus mutans* and *Lactobacilli* spp. In previous study we checked *S. mutans* and *Lactobacillus* sp. CFU counting in saliva source, as well as in biofilm microcosm after 5 days of growing.^26^ The levels of the microorganisms increase in biofilm compared to the saliva source *Streptococcus mutans* (original human saliva: 1.64 x 10^4^ genomes/ µl DNA fraction of saliva, biofilm-PBS: 4.41 x 10^4^ genomes/ µl DNA fraction of biofilm); and *Lactobacilli* spp. (under detection level in saliva; biofilm-PBS: 5.85 x 10^7^ genomes/ µl DNA fraction of biofilm), so the atmosphere is appropriate to induce a cariogenic biofilm formation.^27,28^ They can produce lactic acid and extracellular polysaccharide, which are responsible for the reduction and maintenance of low pH, respectively. At the end of 5 days, the lesion induced in bovine enamel is about 124.7±23.4 µm depth^26^ and the low sucrose levels allow the maintenance of the intact surface layer. This lesion is deeper than a lesion induced in human enamel with 4 and 6 days, which may be due to the differences in the composition of both dental substrates.

Although the lesion in human enamel was lower than those induced in bovine enamel, it was detectable by two methods of analysis: QLF (clinical analysis) and TMR (laboratory analysis). As expected, TMR was more sensitive to detect changes in mineral content and lesion depth than QLF. In fact, TMR is regarded as the gold standard technique to evaluate mineral content, while QLF has the advantage of being employed clinically. However, this study did not present clinical characteristics, so this advantage was not important. Furthermore, typical white spot lesions were produced, with a visible pseudo-intact layer, especially after 4 days of biofilm growth (Figure 2).

This study, in which classical microcosm biofilm models were employed, with growing experimental periods was used to produce WSLs with increasing degrees of mineral loss and depth. There is only one other study in the literature that evaluated mineral loss and depth of lesions formed in enamel under microcosm biofilm for 4, 8 and 12 days. However, the authors employed a model in which the pH of the culture medium was adjusted to 4.5 or 7 (during demineralization and remineralization, respectively). The authors did not find a time-response pattern regarding ∆Z and lesion depth^23^ as we did. In our study, R showed no change in the different experimental periods, which means that the mineral losses were proportional along to the depths. Therefore, the increase of ∆Z was due to the progression of the lesion to deeper layers.

The degree of mineral loss and lesion depth is known to play an important role in mineral diffusion.^8,29^ Lesions with higher ΔZ at baseline have a pronounced decrease in further mineral loss and a concomitant increase in further mineral gain. The decreased further demineralization of lesions with higher ΔZ at baseline may be due to reduced intrinsic solubility caused by previous loss of carbonate and magnesium. On the other hand, the tendency towards remineralization is related to the fact that more porous lesions are more easily remineralized than less porous lesions.^30^ This helps to explain the tendency toward net remineralization of lesions with higher ΔZ at baseline.^29^ Thus, according to our protocol, lesions formed during 8-10 days would tend to remineralize faster than the lesions formed during 2-4 days.

Regarding the lesion depth, shallow lesions are more susceptible to demineralization than deeper ones. This happens because in the latter the dissolved mineral from the deeper portions may reprecipitate during outward diffusion.^8,31,32^ On the other hand, the remineralization rate is lower in deeper lesions as consequence of the longer distance for ionic diffusion before mineral deposition occurs.^8,31^ Thus, if higher degree of demineralization or remineralization is expected, according to our protocol it would be recommended to employ lesions formed during 2-4 days under microcosm biofilm. On the other hand, when lower rates of de- and remineralization are anticipated, it would be better to prepare WSLs formed under microcosm biofilm for 8 –10 days.

In addition to the initial mineral loss of WSLs, its mineral distribution is also important.^4^ In our study, the R values were similar among the groups, which is expected since *in vitro* R is constant over the demineralization period.^33^ Moreover, low-R lesions tend to be more suitable when physiological mineral distribution is required. On the other hand, high-R lesions can better discriminate among the treatments being studied.^8^ The R of natural enamel WSLs has been reported to be around 16,^8^ lower than the values found in the present study, which ranged between 25 and 30, as expected, since the natural lesions are usually developed over a longer period of time. This means that the lesions formed under microcosm biofilm, according with our protocol, might be more appropriate to distinguish among different treatments. Considering these aspects, to compare results from studies having lesions with distinct characteristics at baseline requires caution,^4^ and the results of *in vitro* studies shall be always confirmed clinically.

In conclusion, this study characterized the degree of mineral loss and depth of artificial WSLs formed on human enamel under microcosm biofilm. Our data provide important information regarding the characteristics of the obtained lesions employing different experimental periods. This information can be used in future studies aiming to test caries preventive products *in vitro*.
